# Nanostrategies: The Future Medicine for Fighting Cancer Progression and Drug Resistance 2.0

**DOI:** 10.3390/ijms232214486

**Published:** 2022-11-21

**Authors:** Giovanni Luca Beretta

**Affiliations:** Molecular Pharmacology Unit, Department of Experimental Oncology, Fondazione IRCCS Istituto Nazionale dei Tumori, 20133 Milan, Italy; giovanni.beretta@istitutotumori.mi.it

The attractiveness of the nanomaterials research field has persisted. Since the first description of liposomes in the 1960s and their use as drug delivery systems for the treatment of diseases, several nanotherapeutics have been reported on, leading to the field known as nanomedicine [1]. This novel branch of medicine, which includes theranostics [2], has raised treatment expectations for patients affected by tumors, also offering novel medical options for fighting their multidrug resistance, the primary cause of cancer relapse. The identification of the cellular mechanisms subtending multidrug resistance, as well as possible alternatives to conventional chemotherapy, has become a central goals of cancer research [3]. In this context, the surface decoration of nanovectors with target-directed moieties and their encapsulation with combinations of different drugs (multifunctional nanodevices) have resulted in increased drug accumulation in tumors, the more efficient bypass of biological barriers and, in turn, enhanced activity against drug-resistant tumors [4,5].

The majority of papers published in this Special Issue focus on breast cancer, the most frequently diagnosed tumor in women (11.7% of all cancer cases) and one of the leading causes of death (6.9% of total cancer deaths globally) [6]. Though conventional chemotherapy is not as effective as desired in late-stage metastatic disease, issues concerning its bioavailability, low cellular uptake, drug resistance and adverse toxicity have emerged. In this context, nanomedicine represents a promising alternative to conventional treatment for breast cancer, and the review by Tagde et al. summarizes the state-of-the-art of nanomedicine-based therapeutic interventions that are becoming more widely accepted for improving treatment effectiveness and reducing undesired side effects [7]. Very peculiar is the topic of the review by Serini et al., which recapitulates studies of nanovectors based on nutritional ω-3 polyunsaturated fatty acids for drug delivery in breast cancer [8]. These ω-3 polyunsaturated fatty acids have displayed potent anti-inflammatory and antiangiogenic activities that impair cell growth and reprogram the tumor microenvironment. These nanosystems, loaded with doxorubicin, paclitaxel and 5-fluorouracil, potentiate the in vitro and in vivo activity of antitumor drugs, enhance their accumulation in tumors and minimize drug-induced side effects. The review tackles the antitumor efficacy of these nanoformulations in preclinical in vivo models of ovarian cancer as well, and suggests directions to address still lacking clinical trials.

Nanomaterials hold immense promise for overcoming tumor drug resistance; the articles by Gote et al. and Juan et al. discuss various nanomedicines designed within this scope in breast cancer, including triple negative breast cancer [9,10]. The enhanced selectivity towards cancer cells produced through the use of targeted nanomedicines allows for a guided drug release into tumors, more precise breast cancer diagnoses and the overcoming of multidrug resistance. The focus of Gote et al.’s review is expanded through a discussion on the antitumor potential of nanoparticles encapsulating more than one drug, theranostic nanoparticles, as well as stimuli-sensitive or “smart” nanoparticles. The innovation presented by microRNA-loaded nanodevices and their usefulness in precision-personalized medicine are also discussed. The review by Juan et al. embraces antibody-conjugated nanoparticles and their potential use in clinics. These nanoparticles encapsulate therapeutics and are directed at tumors through conjugation with antibodies. In this way, the chemical structure of drugs is preserved, and the targeted vehicle allows for a controlled drug delivery with reduced toxicity. The article also discusses the application of antibody-conjugated nanoparticles for the treatment of breast cancer subtypes with limited therapeutic options.

A monoclonal antibody (HER2 Affibody-IR700Dye) directed against the receptor HER2 and conjugated with a photosensitizer (IR700Dye), activated by near-infrared light irradiation (near-infrared photoimmunotherapy), is the topic of the manuscript by Yamaguchi et al. [11]. The research aims at expanding the targeted scope toward HER2-positive breast cancer, showing that the combined treatment of HER2-positive cells with the HER2 Affibody-IR700Dye conjugate and trastuzumab-IR700Dye conjugate, which targets two different epitopes of HER2, specifically provokes necrotic cell death, and that this effect is superior to that produced by the administration of the two antibodies alone. The combination has been shown to be very effective against cancer cells expressing low levels of HER2 and on trastuzumab-resistant cells, as well as on brain metastatic cells of breast cancer. Following conjugation with a fluorescent dye, the two antibodies showed interesting in vivo imaging features and specifically identified HER2-positive tumors.

The Special Issue includes two interesting studies focused on photothermal therapy for the treatment of tumors [12,13]. Photothermal therapy, which induces cell death through an increase in temperature (photothermal effect), can be considered an alternative to surgery due to being better tolerated by patients. Carrese et al. propose promising hybrid silica/melanin nanoparticles built around silver seeds and loaded with doxorubicin for an integrated photothermal chemotherapy approach to breast cancer. The empty nanodevice shows photoacoustic contrast, making it useful for imaging. The combined treatment of doxorubicin-loaded nanoparticles and photothermal therapy allows for efficient drug delivery and enhanced cytotoxicity at significantly lower doses than chemotherapeutic treatment alone. The research by Kim et al. is a numerical analysis of heat transfer inducing apoptosis in tumor tissue under different heating conditions mediated using photothermal therapy induced through a gold nanorod treatment. The study also reveals the optimal conditions for maximizing the efficacy on tumor tissue while minimizing damage to surrounding tissues following the heat treatment. Based on the application of the Monte Carlo method on a multilayered skin structure containing squamous cell carcinoma, numerical correlations are reported between the laser intensity and the volume fraction of the gold nanorods administered and the effective apoptosis induction.

The drug resistance of lung cancer and nanostrategies considered to overcome this drawback are the topics of the review article by Haider et al. [14]. The identification of the mechanisms of drug resistance in lung cancer patients is a central theme in the management of this inconvenience. The review summarizes the advances in nanotechnology that have resulted in the development of targeted and multifunctional nanoscale drug constructs that have proved efficacy in overcoming the drug resistance of lung cancer in vitro. Additionally, the authors overview clinical trials containing nanocarrier-based drug delivery systems under evaluation in lung cancer patients. Finally, the article discusses future directions for the clinical application of nanomedicine in the management of lung cancer resistance, including understanding formulation drawbacks and the challenges represented by the various biological barriers, also considering possible novel strategies, including iron- and siRNA-loaded nanoparticles and photodynamic therapy.
